# An audit into the monitoring of off-label antipsychotics in primary care

**DOI:** 10.1192/bjo.2021.569

**Published:** 2021-06-18

**Authors:** Damir Rafi, Javier Ferreiro-Pisos, John Millwood Hargrave, Cristina Losada Pérez

**Affiliations:** 1 South London and Maudsley NHS Foundation Trust; 2 Guys and St Thomas' NHS Foundation Trust; 3 grupo doctor oliveros

## Abstract

**Aims:**

To ascertain whether patients prescribed second generation antipsychotics for off-label indications are being monitored and screened adequately for physical health side-effects.

**Background:**

The prevalence of off-label antipsychotic use has increased significantly over recent decades. Common off-licence uses include dementia, post-traumatic stress disorder, adjunctive treatment for unipolar depression and personality disorders. Recent studies have demonstrated that up to 65% of antipsychotic prescriptions are now off-label. Since the metabolic side-effects of second-generation antipsychotics are well-established, guidelines have emphasised the need for active, routine physical health screening of all individuals taking these drugs. However, there have been few studies or reviews which have specifically investigated screening rates of individuals receiving antipsychotic medications for off-licence indications.

**Method:**

An audit of patients taking second-generation antipsychotics for off-label indications, under the caseload of Neighbourhoods 1, 3 and 4 of Lewisham Assessment & Liaison team, was conducted. After isolating individual patients fulfilling inclusion criteria, patient investigation documents were requested from relevant GP practices. 40 patients were isolated in total, and data were successfully collected in 60% (n = 24). Data were collected via a proforma. This consisted of patient information, indications for antipsychotic use, and each variable to be monitored. The audit standard used was the recommendations of the 12th Maudsley guidelines. Data were then entered into SPSS and analysed.

**Result:**

The most common reasons for off-label antipsychotic prescribing were Emotionally Unstable Personality disorder (42%, n = 10) and depression (29%, n = 7). Findings demonstrated that 54% (n = 13) of patients audited had ‘basic’ blood screening (FBC, U&E, LFTs), however glucose (38%, n = 9), Prolactin (13%, n = 3), and Creatine Kinase (0%, n = 0), and monitoring was less frequent. 0% (n = 0) were completely monitored as per audit standard.

**Conclusion:**

Primary care monitoring of off-label antipsychotics is unsatisfactory, with no patients having a complete set of investigations. Reasons for this are unclear at this stage, however based on initial discussion with GP surgeries, may be due to lack of education regarding screening investigations, patients lost between primary and secondary care services, and a lack of clarity regarding responsibility and designated roles. This audit will be expanded to also include patients from Neighbourhood 2 of the Lewisham Assessment & Liaison team. A more detailed investigation will be conducted into the barriers to physical health screening, such that a targeted intervention can be implanted.

